# Evolutionary history of the endangered Sanje mangabey (*Cercocebus sanjei*) in the Udzungwa Mountains, Tanzania: inferences from phylogeography and historical niche modelling

**DOI:** 10.1007/s10329-026-01255-2

**Published:** 2026-04-10

**Authors:** Christina Lynette Paddock, Maria Joana Ferreira da Silva, Gráinne Michelle McCabe, David Fernández, William Scott McGraw, Michael William Bruford

**Affiliations:** 1 School of Biosciences, Cardiff University, Cardiff, UK; 2 Wilder Institute, 1300 Zoo Road NE, Calgary, AB T2E 7V6 Canada; 3 Bristol Zoological Society, Bristol, Avon, United Kingdom; 4 Department of Anthropology and Archaeology, University of Calgary, Calgary, Canada; 5 Department of Anthropology, The Ohio State University, 4042 Smith Laboratory, 174 W. 18th Avenue, Columbus, OH 43210 USA

**Keywords:** Ecological niche modelling, Genetic differentiation, Afro-montane, East Africa, Management units (MUs), Evolutionarily significant units (ESUs)

## Abstract

Understanding whether a species’ distribution results from recent and/or anthropogenic events or ancient vicariant factors is critical for conservation planning. The Endangered Sanje mangabey (*Cercocebus sanjei*), endemic to Tanzania’s Udzungwa Mountains, is currently divided into two populations located approximately 100 km apart. These represent distinct evolutionary lineages that diverged around 0.77 million years ago (MYA). We aimed to investigate i) the phylogeographic history and recent changes in size and range of the two Sanje mangabey populations, and ii) whether lack of suitable habitat between populations and consequent difficulty to disperse may have influenced the 0.77 MYA divergence time. We used 64 mitochondrial control region sequences obtained from non-invasive DNA. The probability of suitable habitat across Tanzania and the Udzungwa Mountains was modelled at three time points: Mid-Holocene (6,000 YA), Last Glacial Maximum (22,000 YA), and the Last Interglacial period (120,000–140,000 YA). We found six haplotypes, clustered into two haplogroups. Significant differentiation was estimated between populations, which show no evidence for recent range expansion or contraction. The ecological niche modelling revealed fluctuating extents of suitable habitat across southern Tanzania. Large genetic differentiation between populations may have been influenced by a general trend in aridification in East Africa across the last 40,000 years, resulting in a shift of montane forests to gradually higher elevations. Intermediate populations may have become extinct as suitable habitat retracted, leaving relict populations with relatively stable demographic histories ancestral to the present-day populations. This study supports their preliminary designation as separate evolutionary significant units, a conclusion with conservation management implications.

**Supplementary Information:**

The online version contains supplementary material available at https://doi.org/10.1007/s10329-026-01255-2.

## Introduction

The Sanje mangabey (*Cercocebus sanjei*) is an Endangered primate that is endemic to the Udzungwa Mountains, Tanzania (McCabe et al. 2019). The species is found in only two forest fragments: Mwanihana in the Udzungwa Mountains National Park and the Uzungwa Scarp Nature Reserve (hereafter Udzungwa and Uzungwa Scarp, respectively). The Uzungwa Scarp is currently isolated and no viable ecological corridor exists between it and neighbouring forests (WWF Tanzania Programme Office 2007). The two forests in which the populations are found are ~ 100 km apart and are separated by habitats thought to be unsuitable for the species (Rovero et al. 2014).

This division into two small and isolated populations suggests that the species is at great risk of potentially negative stochastic demographic and genetic processes, such as genetic drift and inbreeding depression, and loss of genetic diversity and adaptive potential (Ferreira da Silva and Bruford 2017; Frankham 2010). It is currently unknown when these two forests became isolated, with one study suggesting ‘some have probably been separated for > 100 years’ (Struhsaker et al. 2004). Based on a study using two nuclear and two mitochondrial markers, the two Sanje mangabey populations were estimated to be representative of two evolutionary distinct lineages (Paddock et al. 2026). The lineages were estimated to have diverged 0.77 million years ago [(MYA), 95% confidence intervals (CI): 0.43–1.15 MYA)] and were suggested to be considered candidates to evolutionary significant units (ESUs, Moritz 1994) pending more evidence (Paddock et al. 2026).

To date, there have been no attempts to determine the phylogeographic structure of the Sanje mangabey populations, or to investigate the underlying causes of the genetic divergence between the two populations (Paddock et al. 2026). However, as with the divergence time estimated between populations, evidence from genetic studies of other animals in the Udzungwa Mountains have found significant genetic differentiation between forest blocks with isolation of fragments indicating a relatively ancient timescale (i.e., thousands or millions of years ago, KYA/MYA). One study found evidence for mitochondrial DNA introgression between some populations of the grey-faced sengi (*Rhynchocyon udzungwensis*) and the parapatric and more widespread chequered sengi (*R. cirnei reichardi*), (Lawson et al. 2013). However, nuclear loci showed clear monophyly between species, suggesting introgression occurred on a historical timescale, coinciding with interglacial climate cycles altering each species’ distribution (Lawson et al. 2013). A study of the landscape genetics of the sympatric and endemic Udzungwa red colobus (*Piliocolobus gordonorum*) across five forests, including Mwanihana and the Udzungwa Scarp, found that, although neutral genetic diversity did not differ within fragments, there was genetic differentiation between fragments (Ruiz-Lopez et al. 2016). It was also determined that variables such as proximity to human settlements and human-caused wildfires (Dinesen et al. 2001), were shaping the species’ genetic structure. Nevertheless, results suggested that genetic differentiation may still be a product of ancient fragmentation and recent human activity was responsible for maintaining existing barriers to dispersal (Ruiz-Lopez et al. 2016). The Uzungwa Scarp red colobus population was the most isolated, had the highest differentiation and the highest number of unique alleles, and lack of migration for several generations (Ruiz-Lopez et al. 2016). Therefore, it is possible that other sympatric primate species with relatively long generation times, including the Sanje mangabey, have a similar history.

**Fig. 1:**
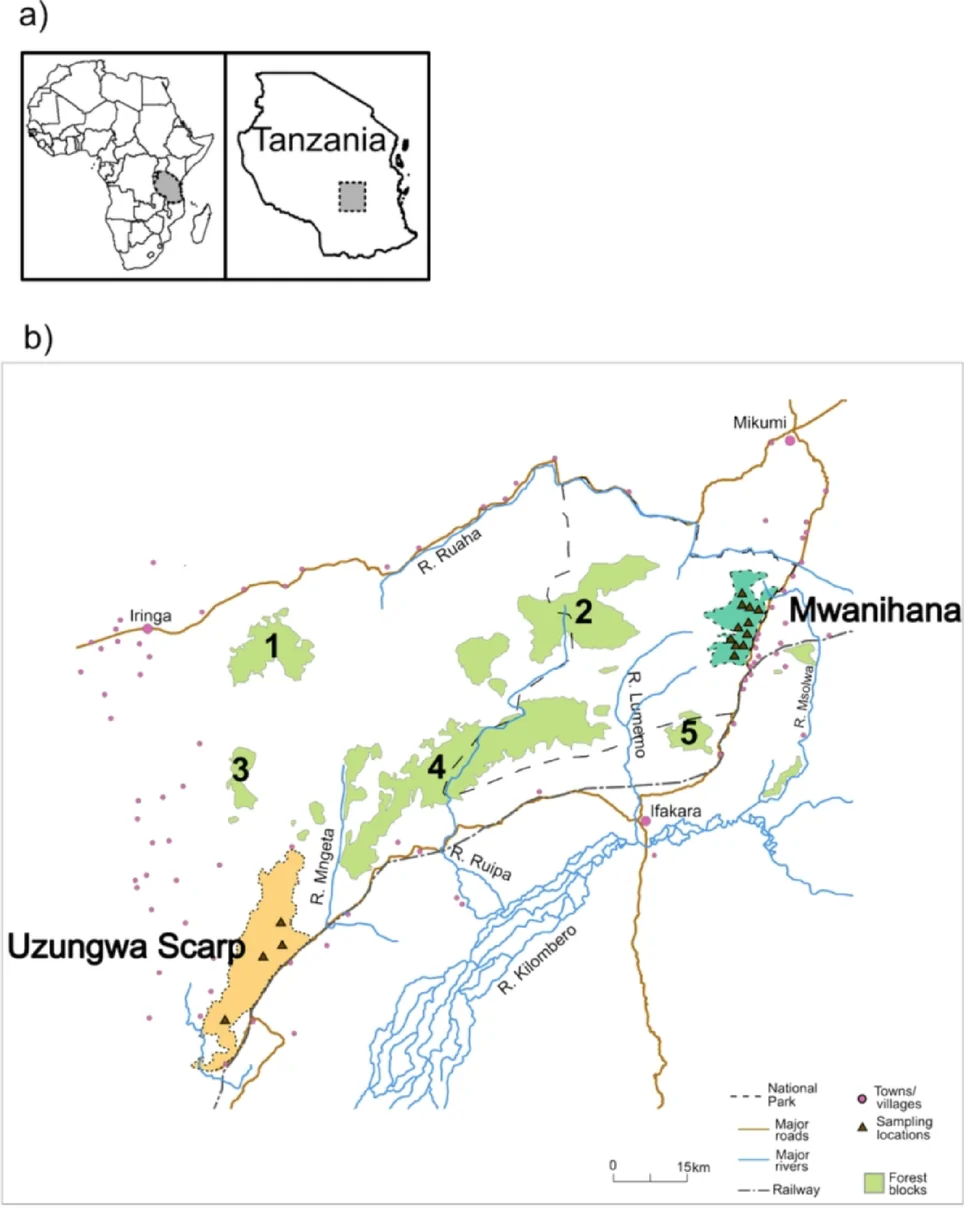
**a** Location of Tanzania and the Udzungwa Mountains **b** a map showing the two forests where the Sanje mangabey is found: Mwanihana forest (in dark green) and the Uzungwa Scarp (in orange). The locations where faecal samples were collected are highlighted by purple triangles. The figure also depicts the limits of the national parks (black discontinuous line), major roads (brown continuous line), major Rivers (blue continuous line) and Railways. Other forest blocks are represented in light green: 1 – Kisinga-Rugaro, 2 – Luhombero / Ndundulu, 3 – New Dabaga, 4 – Matundu, 5—Nyanganje

The Sanje mangabey occupies an elevation range between 290 and 2000 m above sea level (a.s.l.) (McCabe et al. 2019). They are flexible in their habitat use, successfully making use of primary and secondary forest, and elephant-disturbed shrubland (McCabe et al. 2013). Throughout the Udzungwa Mountains, other forests exist within this elevation range with similar habitat composition (Marshall et al. 2010) and are inhabited by primate species that are sympatric with the Sanje mangabey in Mwanihana and the Uzungwa Scarp (Rovero and Luca 2007). Therefore, it is likely that ancient Sanje mangabey subpopulations existed in other forest blocks within the region that have since become extinct, with the original description of the species suggesting this may have included Kilombero Valley lowland forests (Homewood and Rodgers 1981), an area located south east of both extant populations along the River Kilombero (Fig. 1). This extended range may have included two neighbouring forests to Mwanihana (< 30 km): Nyanganje (41.9 km^2^; 350–1,038 m) and Luhombero/Ndundulu (230.6 km^2^; 1,105–2,520 m), both home to the Udzungwa red colobus, black-and-white colobus (*Colobus angolensis palliatus*) and the Syke’s monkey (*Cercopithecus mitis* sp.), with the yellow baboon (*Papio cynocephalus*) and vervet monkey (*Chlorocebus pygerythrus *) also present in Nyanganje and the kipunji (*Rungwecebus kipunji*) in Luhombero (Fig. 1). These forests are considered to be minimally impacted by human activities (Marshall 2007; Marshall et al. 2010) but have previously been targets for logging (Dinesen et al. 2001; Rovero and Marshall 2005) and hunting (Jones et al. 2005). Similarly, neighbouring the Uzungwa Scarp (< 30 km), forests within this elevation range include Kisinga-Rugaro (116.2 km^2^; 1,627–2,322 m) and New Dabaga/Ulangambi (40 km^2^; 1,764–2,081 m), with the black-and-white colobus, Syke’s monkey, and vervet monkey present in both forests, and the Udzungwa red colobus in New Dabaga (forest size and elevation data from Marshall et al. 2010). No endemic species of primate are found in Kisinga-Rugaro; however, the Udzungwa red colobus has been reported to be present previously (Lovett and Pocs 1993). Both of these forests have been subject to high anthropogenic activity, especially extensive logging in the 1970s (Dinesen et al. 2001). It is therefore possible that the Sanje mangabey could have inhabited these forests and become extirpated with habitat degradation and/or hunting (Rovero et al. 2009).

Ecological niche modelling (ENM) could be biogeographically informative by identifying habitats similar to those at Mwanihana and the Udzungwa Scarp but that are not currently occupied by the Sanje mangabey. ENM is a mathematical tool used to estimate the probable area of suitable habitat, and a predicted distribution can be modelled using the known distribution or presence data of species and environmental parameters (Thuiller 2024). Moreover, combining estimated paleodistributions, projected from historic bioclimatic estimates with genetic structure from current sampling can provide a spatial perspective on a species’ demographic history and distribution (Khanal et al. 2018). ENM is also an invaluable tool for conservation as it can provide information on the importance of environmental variables and potential distribution of species to target surveys and potential areas for conservation (e.g., western chimpanzees, *Pan troglodytes verus*, Fitzgerald et al. (2018) and the Yunnan snub-nosed monkey *Rhinopithecus bieti* in Laojun Mountain National Park, China, Liu et al. (2020)), and assist in optimal land use planning to preserve biodiversity by local stakeholders (Gregory et al. 2012; Zhang et al. 2012).

Determining whether the current distribution of a species is a function of recent and/or anthropogenic-related events, versus more ancient and vicariant factors, is important for the creation of appropriate conservation policies (Radespiel and Bruford 2014). Significant divergence in genetic structure between populations would substantiate a claim for those populations to be classified as separate management units (MUs) or as evolutionarily significant units (ESUs) (e.g., Moritz 1994). The identification of such units should then be incorporated in conservation action plans to ensure genetic diversity is adequately protected and included in assessments of measures such as taxonomic designation, translocation and landscape restoration. Such studies have relied on the use of mitochondrial DNA (mtDNA) as a genetic marker for identifying recent and historic phylogeographic patterns (Sunnucks 2000; Bertola et al. 2024), which has resulted in a large database of sequences available for a wide range of species. MtDNA presents advantages when compared to nuclear DNA in these contexts, namely (i) a faster evolution rate, in particular of the D-loop region, which means that more recent changes (i.e., historical timeframes) are better detected, (ii) a smaller effective population size (of approximately 25%), meaning mtDNA is more affected by processes such as genetic drift; (iii) easy amplification through Polymerase Chain Reaction (PCR) from non-invasive collected samples (e.g., hair or faeces), which are preferred in studies focussing in cryptic and threatened species. For example, two ESUs were identified in muriquis in the Brazilian Atlantic Forest (northern: *Brachyteles arachnoides*; and, southern: *B. hypoxanthus*) using a fragment of the mtDNA control region (Chaves et al. 2019). In this case, conservation strategies were tailored for each population, rather than following the monotypic classification *B. arachnoides* and applying the same actions to both populations (Chaves et al. 2019).

This study aimed to characterise the phylogeographic structure, mitochondrial genetic diversity and recent demographic history of the Sanje mangabey using a fragment of the mtDNA control region, obtained from samples collected from across the distribution of both populations: the Mwanihana Forest and the Uzungwa Scarp (Fig. 1). We used ENM to estimate the extent, location and temporal dynamics of possible suitable habitat for the Sanje mangabey over the last 140,000 years to assess whether lack of suitable habitat and consequent inability to disperse between populations may have influenced the 0.77 MY divergence time (Paddock et al. 2026). We hypothesised that the phylogeographic structure of the Sanje mangabey populations would reflect the ancient timescale of divergence and therefore would show significant mitochondrial differentiation between populations, reflecting evolutionary distinct lineages requiring independent conservation action. Further, combining the demographic history with the ENM, we hypothesised that patterns in effective population size would follow trends of estimated suitable habitat extent. Conservation management recommendations based on results from genetic data analyses and ENMs are included, with the objective to complement the conservation priorities outlined in the International Union for Conservation of Nature’s (IUCN) *Cercocebus and Mandrillus Conservation Action Plan* (Dempsey et al. 2024).

## Methods

### Study area and sampling

The study area includes the current distribution of the Sanje mangabey in south-central Tanzania in two forests within the Udzungwa Mountains: Mwanihana Forest (7°40’–7°57’S, 36°46’–36°56’E) within the Udzungwa Mountains National Park, and the Uzungwa Scarp Nature Reserve (8°14’-8°32’S, 35°51’-36°02’E) (Fig. 1).

We collected faecal samples opportunistically from unidentified individuals, across 28 unique locations coinciding with a population acoustic survey of the species within both forests (described in Paddock et al. 2020) (Fig. 1). Samples were collected while following groups located from vocalisations. Sampling locations were selected using a criterion to avoid repeated sampling of the same mangabey group at multiple locations (Paddock et al. 2020). In order to minimise the probability of repeatedly sampling an individual, at each unique sampling location, we only collected samples at minimum of 2 m apart from another sample and selected samples that could be confidently attributed to distinct individuals based on different color and shape (Paddock et al. 2026). We stored samples following the two-step method, a protocol in which samples are immersed in 96% ethanol for a minimum of 24 h and then stored in desiccating silica gel until DNA extraction (Roeder et al. 2004). Samples were transported for analysis to the School of Biosciences, Cardiff University, UK.

### DNA extraction, amplification, sequencing and data quality control

We extracted DNA from the outer surface of those faecal samples and used a QIAamp Fast DNA Stool Mini Kit (Qiagen, UK) following a modified manufacturer’s protocol (Paddock et al. 2026).

We attempted the amplification by PCR of a 580 base pairs (bp) fragment of the mtDNA CR, which spans sections of both the hypervariable regions I and II (HVRI: ~ 350 bp; HVRII: ~ 230 bp) (Paddock et al. 2026). We sequenced from two overlapping primers using PCR (Paddock et al. 2026, Table S1, online material). We sequenced PCR products bi-directionally using Sanger technology using either the Eurofins PlateSeq Service (Ebersberg, Germany) or the Centre for Molecular Analysis (CTM; CIBIO, Portugal) sequencing service. We inspected the chromatogram for each sequence manually using Geneious (v4.8.5) to trim forward and reverse ends and verify the quality of base calls. We trimmed sequences to the length of the shortest sequence, resulting in a 423 bp fragment for analyses. Several precautions were taken to verify the co-amplification of nuclear mitochondrial inserts (NUMTS), following recommendations of Bensasson et al. (2001): (i) sequence chromatograms were inspected for double peaks that would indicate the presence of sequences co-amplified by PCR and which could represent contaminations or NUMTS, (ii) only a single PCR amplicon of the correct length was present, (iii) any single base extreme variants found during the alignment of sequences were verified, and (iv) four independent primers amplifying overlapping fragments were used, with the overlapping sequences matching from separate PCRs (Table 1). We excluded low quality sequences from the final dataset. Each sequence was submitted to BLAST (Basic Local Alignment Search Tool; https://blast.ncbi.nlm.nih.gov/Blast.cgi) to search against existing sequences in GenBank. We only retained samples showing > 95% of percentage identity to *Cercocebus* voucher sequences.

**Table 1:** Population mitochondrial genetic diversity indices for each Sanje mangabey (*Cercocebus sanjei*) population individually and for the overall population, using a 423 bp fragment of the control region

Population	*n*	*N*_hap_	*h* (± SD)	*S*	π (± SD)	k
Mwanihana	36	2	0.056 (± 0.052)	1	0.00013 (± 0.00012)	0.056
Uzungwa Scarp	28	4	0.664 (± 0.055)	5	0.00417 (± 0.00041)	1.756
Overall	64	6	0.642 (± 0.051)	22	0.022 (± 0.011)	9.273

*n* = sample size; *N*_hap_ = number of haplotypes; *h* = haplotype diversity, *S* = polymorphic sites; π = nucleotide diversity; k = mean pairwise differences

### Genetic diversity and phylogeographic history

We estimated the number of polymorphic sites, number of haplotypes, haplotype diversity and nucleotide diversity using DnaSP v 6.12.01 (Rozas et al. 2017). To estimate the relationship between haplotypes, we built a TCS network of haplotypes using PopART v1.7 (Leigh and Bryant 2015).

We tested for the presence of genetic structure in the population using an Analysis of Molecular Variance (AMOVA) and the fixation index (*F*_*ST*_), which was computed using pairwise differences with 10,000 permutations in ARLEQUIN 3.5.2.2 (Excoffier and Lischer 2010).

The inference of the demographic history when differentiated populations are analysed as a single unit may produce an erroneous signal of a demographic change (Heller et al. 2013). Thus, because populations were previously found to be representative of ancient lineages (Paddock et al. 2026) and significant differentiation was found between populations in this study (see Results), the demographic history was inferred for the two populations independently. We investigated the demographic history of the populations using the neutrality tests Tajima’s *D* (distribution of segregating sites) and Fu’s *F*_*s*_ (haplotype distribution) statistics, that were carried out in ARLEQUIN 3.5.2.2 (Excoffier and Lischer 2010) using 10,000 simulations for each population individually. Positive values of *D* and *F*_*s*_ would indicate the loss of rare alleles following population contractions, whereas significant negative values would indicate a recent population expansion (Ramírez-Soriano et al. 2008). Statistical significance was considered at p < 0.05.

We inferred changes in effective population size using the Coalescent Bayesian Skyline model (BSP) in BEAST v2.6.0 (Bouckaert et al. 2019) for each population individually. We assessed the most probable evolutionary model using jModelTest 2.1.10 (Darriba et al. 2012) and selected the HKY + G substitution model using the BIC value criterion. We carried out the BSP analyses using the mtDNA control region clock rate (0.0209 mutations/site/million years; strict clock model) estimated in the BEAST Calibrated Yule model in (Paddock et al. 2026) which is appropriate for comparing sequences from different species. We ran each model with a 100 million chain length, sampling every 1000 runs with a 10% burn in and modelled with five groups. We checked convergence in the model (ESS values > 200) in TRACER v1.7.1 (Rambaut et al. 2018).

### Ecological Niche modelling

We used ENM maximum entropy framework to calculate the probable distribution of the Sanje mangabey. This model minimises the relative entropy between probability densities from presence and environmental data (see Elith et al. 2011). We modelled ecological niches using the software Maxent v 3.4.1 (Phillips et al. 2006).

We defined two study areas within the boundaries of 27.5–42°E, 0–12°S—Tanzania and southern Kenya and the Udzungwa Mountain region, to estimate areas of probable occurrence at two scales. We used presence data only, which was collated from geographic coordinates of vocalisations detected during a population survey (Paddock et al. 2020). We removed duplicate records and spatial autocorrelation by filtering the presence points. We placed a 2.5 arc-minute resolution grid on each forest—which reflects the largest cell size from the downloaded bioclimatic layers—and randomly selected a single presence point from each cell using R (R Core Team 2017). The procedure reduced the original 856 presence co-ordinates to 18 presence points (Mwanihana: *N* = 10; Uzungwa Scarp: *N* = 8), which were used as the occurrence points in the Maxent model. Presence points were approximately 1 km from each other.

We downloaded nineteen ‘near current’ (1960–1990) bioclimatic variables from WorldClim Version 1.4 (http://worldclim.org/) at 30 s resolution (Table 2, online material). We aimed for the highest resolution available at the time of this study. Layers were cropped to the same extent of the study areas and were projected to the same co-ordinate reference system (WGS84:4326) and resampled to 30 s using the *raster* package in R (R Core Team 2017).

**Table 2:** Analysis of Molecular Variance (AMOVA) of genetic differentiation among and within the two Sanje mangabey (*Cercocebus sanjei*) populations for both haplotypic and locus-by-locus distance matrices

Sources of variation	d.f	Sum of squares	Variance components	Percentage of variation
Among populations	1	267.30	8.47	95.49
Within populations	62	24.79	0.40	4.51
Total	63	292.09	8.87	

We used a Pearson’s correlation (*r*) test in R (R Core Team 2017) to measure the correlation between variables. We retained one variable from highly correlated dyads (-0.7 < *r* > 0.7), prioritising the broader one (i.e., annual mean temperature over specific seasonal variables). This resulted in removing 13 of the 19 layers from the analyses (Table S2, online material). The variables used in the ENMs were: Bio1 Annual mean temperature, Bio2 Mean diurnal range: (Mean max. temp – min. temp), Bio3 Isothermality: temperature variability from day to night and between seasons—(Bio2/Bio7) *100, Bio12 Annual precipitation, Bio14 Precipitation of driest month and Bio18 Precipitation of warmest quarter.

We downloaded the retained six variables as estimated for three paleo-bioclimatic scenarios from WorldClim 1.4 and used them for projections of estimated suitable environmental conditions. This included the Mid Holocene (MH: ~ 6000 YA; 30 secs), the Last Glacial Maximum (LGM: ~ 22,000 YA; 2.5 min), and the Last Inter-Glacial (LIG: ~ 120,000–140,000 YA; 30 secs; Otto-Bliesner et al. 2006).

We used the ENMeval R package (Kass et al. 2021) to estimate the best fitting model between combinations of the regularisation multiplier and feature classes. We used the following combinations of the five feature classes (linear: L, quadratic: Q, product: P, hinge: H, and threshold: T): ‘L’, ‘LQ’, ‘H’, ‘LQH’, ‘LQHP’ and ‘LQHPT’ and ten regularisation multiplier values (1–10). The best model would be estimated where ΔAIC = 0.

We used the model’s Area Under the Receiver Operating Characteristic (AUC) curve as a measure of the model performance. The AUC in a presence-only model indicates whether the probability values of the model estimated for presence points are higher than those of the background points. An AUC value of 0.5 for a model indicates random prediction and values closer to 1 indicate better model performance (Townsend 2007). We ran the model, with the features and regularisation multiplier determined to be most appropriate using the cross validate replicate run type, repeated for 25 replicate runs, and with a maximum of 10,000 background points. The cross-validation replicate method is considered better for small datasets as it uses all the data for validation in a series of ‘folds’ with the data split into equal size groups (Phillips 2017). We set the Maxent parameters to logistic output and 5,000 iterations for each simulation.

We generated response curves for the current model to estimate the contribution of each predictor variable on the probability of presence. We estimated Jackknife of regularised training gain (i.e., the estimated predictive power) for each predictor variable to evaluate the contribution of each parameter to (i) the species presence singularly, (ii) when acting as the only variable (training gain) and (iii) when individually removed from the full mode (permutation importance).

To determine probable habitat suitability for Sanje mangabeys, we used a minimum training presence threshold (MTP). In this method, the values from areas estimated to be the lowest probability of suitable conditions set the minimum threshold level. We chose this threshold method as a conservative method for estimating habitat suitability, using the data within the model to establish the thresholds, rather than using fixed subjective values (Liu et al. 2005).

## Results

### Genetic diversity and phylogeographic history

**Fig. 2:**
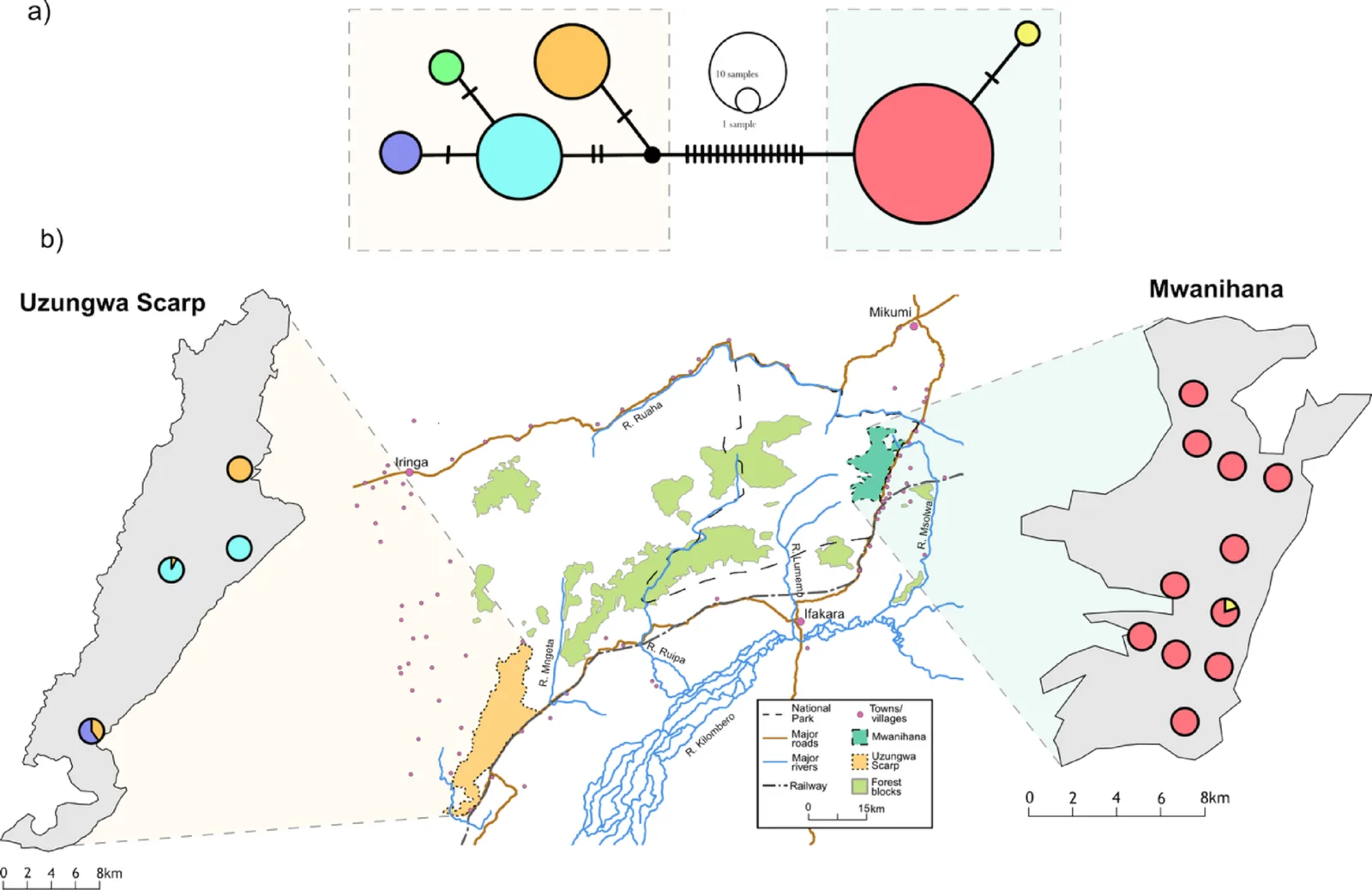
Genetic diversity and phylogeographic structure inferred using a 423 bp fragment of the mitochondrial control region fragment for the Sanje mangabey (*Cercocebus sanjei*) across the forests blocks in the Udzungwa Mountains, Tanzania. **a** a TCS haplotype network showing the relationship between the six haplotypes that were found from 64 sequences; (left) four in the Uzungwa Scarp (n = 28, within a light orange square) and (right) two in Mwanihana (n = 36, within a light green square). Each haplotype is represented by a circle and the size of the circle represents the frequency of each haplotype (please see scale representing 10 and 1 sequences). Small lines represent substitutions between haplotypes. **b** The sampling location of the haplotypes within each park (on the left: Uzungwa Scarp and on the right: Mwanihana). The colours of haplotypes correspond between each figure **a** and **b**.

We obtained 64 Sanje mangabey mtDNA control region sequences sampled at 15 geographically independent sites (Fig. 2) (Mwanihana Forest: *N* = *36* sequences from 11 sites, Uzungwa Scarp: *N* = *28* sequences from 4 sites). We found no evidence for the presence of NUMTs.

We found a total of 22 segregating sites (all transition substitutions) and six haplotypes (Fig. 2, Table 1). Haplotype and nucleotide diversity are higher in the Uzungwa Scarp than Mwanihana (Table 1), with two haplotypes found in Mwanihana and one segregating site; and four haplotypes in Uzungwa Scarp, with five segregating sites (Table 1).

The haplotype network shows two haplogroups, represented by Mwanihana and Uzungwa Scarp, separated by 16 substitutions (Fig. 2). Mwanihana haplotype network shows a frequent and central haplotype connected by one mutation difference to a rare haplotype, harboured by one individual (Fig. 2). For Uzungwa Scarp, the network pattern shows two more frequent haplotypes, connected by three mutation differences, with one of these haplotypes connected to two less frequent haplotypes by one substitution to each (Fig. 2). No shared haplotypes were found between the two populations. The populations are significantly differentiated with 95.49% of nucleotide variation among populations, and only 4.51% within populations (F_ST_ = 0.95, p < 0.001; Table 2).

Using Tajima D and Fu Fs, no significant evidence for demographic expansion or contraction was found for Mwanihana (*N* = 36 sequences: *D* = -1.33, p > 0.10; F_S_ = -1.36, p > 0.10) or the Uzungwa Scarp (*N* = 28 sequences: *D* = 1.04, p > 0.10; F_S_ = 1.914, p > 0.10). Similarly, the BSP for each population shows female effective population size to have been stable in the recent past in both populations (Fig. 1S Online material). The estimated mean female effective population size for current populations is similar for both populations with Mwanihana estimated 0.138 breeding females and Uzungwa Scarp 0.139 breeding females. Although not significant, the Uzungwa Scarp shows an increasing trend in female effective population size over the time sampled, rising from 0.085 at the Last Glacial Maximum to 0.101 at the Mid-Holocene to 0.139 at present (Fig. 1S online material).

### Ecological Niche modelling

After model evaluation, we found an optimal solution using linear, quadratic and hinge feature classes and a regularisation multiplier of 4 to be the best fitting model (AICc = 451.89; ΔAIC = 0). The mean AUC of the Maxent model from averaging the results from 25 runs using the cross validate method was 0.976 ± 0.012, which indicated the data fitted closely with the estimated distribution.

The variables with the greatest contribution to the model were isothermality (bio3; 38.8%), the precipitation during the driest month (bio14; 38.5%), and annual precipitation (bio12; 19.3%; Table 3). The variable for precipitation of the driest month (bio14) had the greatest training gain when used by itself within the model and the overall model training gain decreased most when it was removed, indicating that the variable contains the most information not available in other variables (Fig. 2S online material). It was estimated that precipitation of the driest month had the greatest permutation importance (bio14; 52.4%), followed by isothermality (bio3; 36.4%). The other variables had lower estimated importance (Table 3).

**Table 3:** The estimates of contributions to the Maxent model by each variable

Variable		Contribution (%)	Permutation importance
Bio3	Isothermality	38.8	36.4
Bio14	Precipitation during the driest month	38.5	52.4
Bio12	Annual precipitation	19.3	5.5
Bio1	Annual mean temperature	2.1	4.5
Bio2	Mean diurnal range	1.2	1.2
Bio18	Precipitation of warmest quarter	0	0

**Fig. 3:**
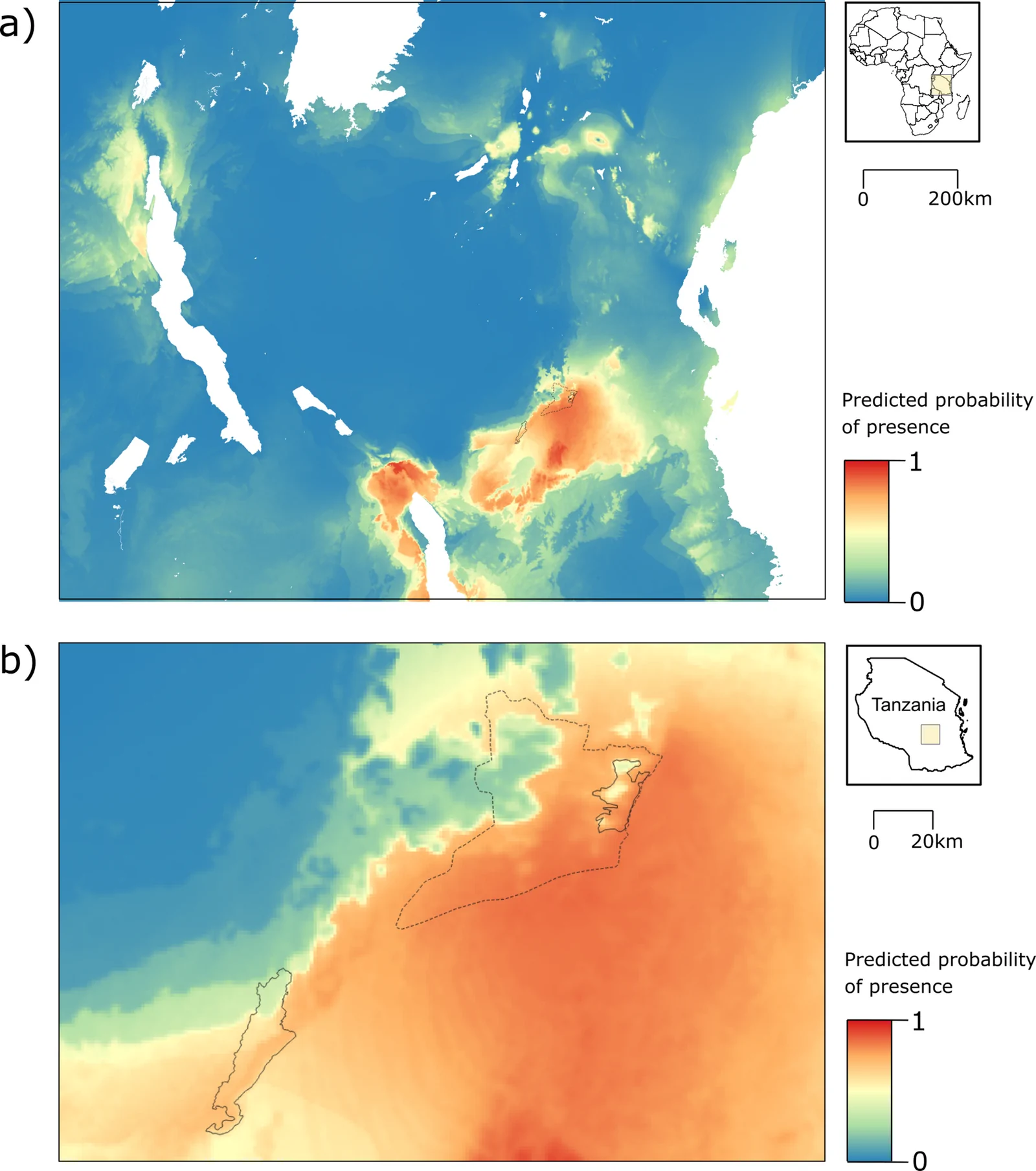
Estimated probability of habitat suitability for the Sanje mangabey (*Cercocebus sanjei*) within the Udzungwa Mountains based on near current (1960–1990) conditions across **a**) Tanzania and **b**) the Udzungwa Mountains

For habitat suitability and presence probability to be estimated over 0.7 (high suitability) in the response curves for the top three contributing variables, isothermality was in the lower range of values in the model (< 63; Fig. 2S, online material), a moderate range of precipitation of the driest month (~ 3–11 mm; Fig. 2S, online material), and high annual precipitation (> 1300 mm; Fig. 3S, online material).

**Fig. 4:**
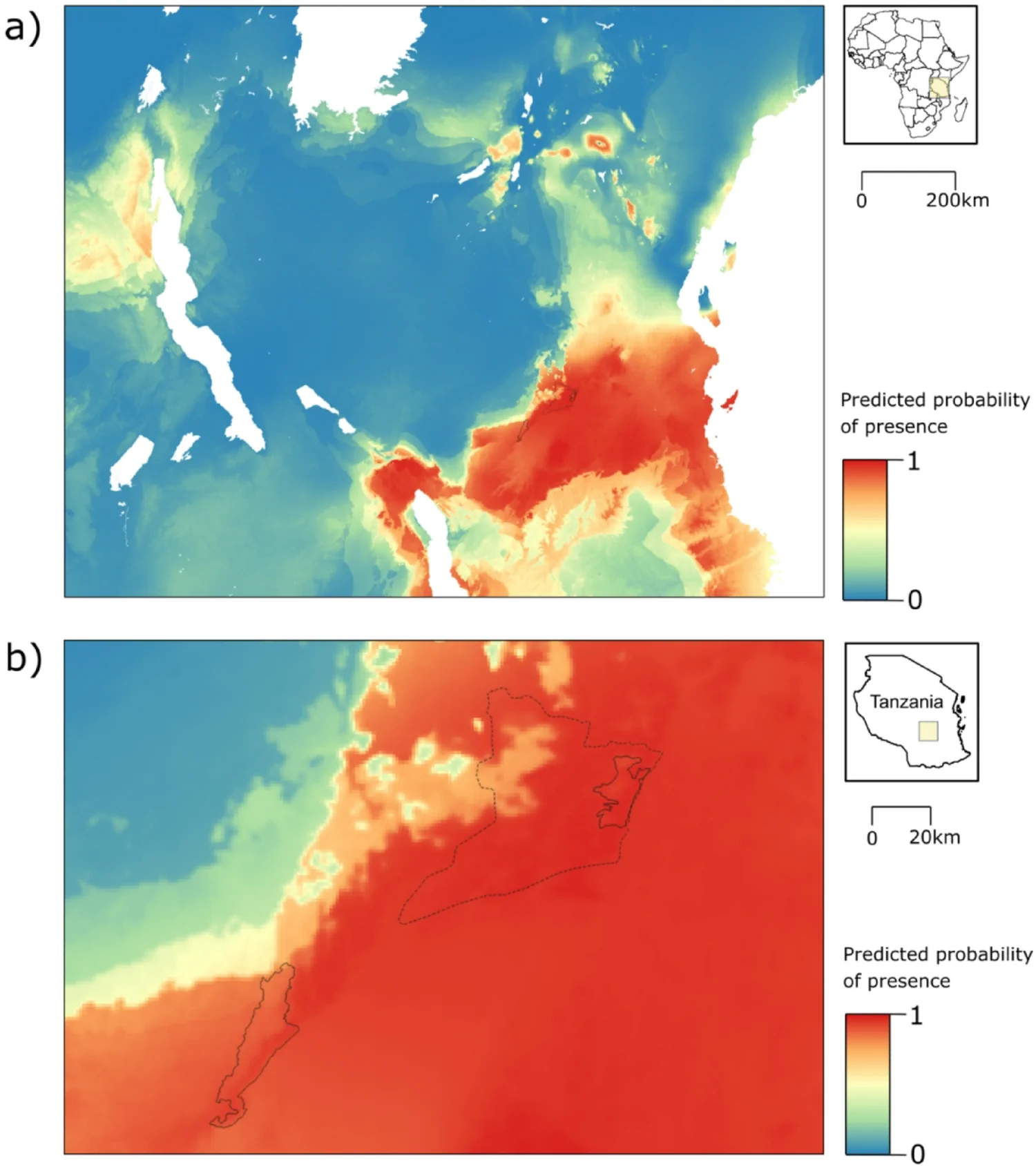
Estimated probability of habitat suitability for the Sanje mangabey (*Cercocebus sanjei*) within the Udzungwa Mountains during the Mid-Holocene (~ 6,000 YA) across **a**) Tanzania and **b**) the Udzungwa Mountains

**Fig. 5:**
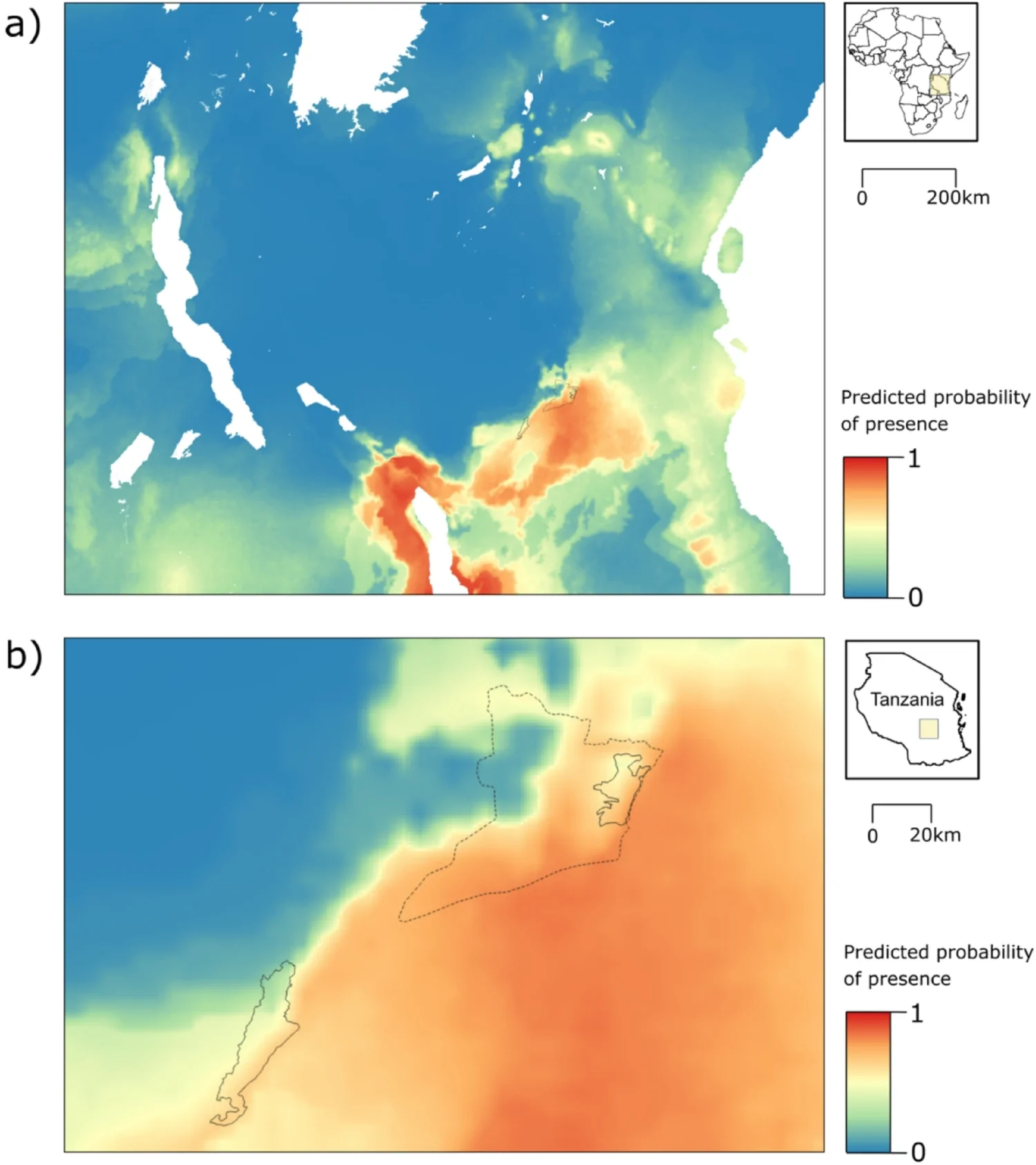
Estimated probability of habitat suitability for the Sanje mangabey (*Cercocebus sanjei*) within the Udzungwa Mountains during the Last Glacial Maximum (~ 22,000 YA) across **a**) Tanzania and **b**) the Udzungwa Mountains

**Fig. 6:**
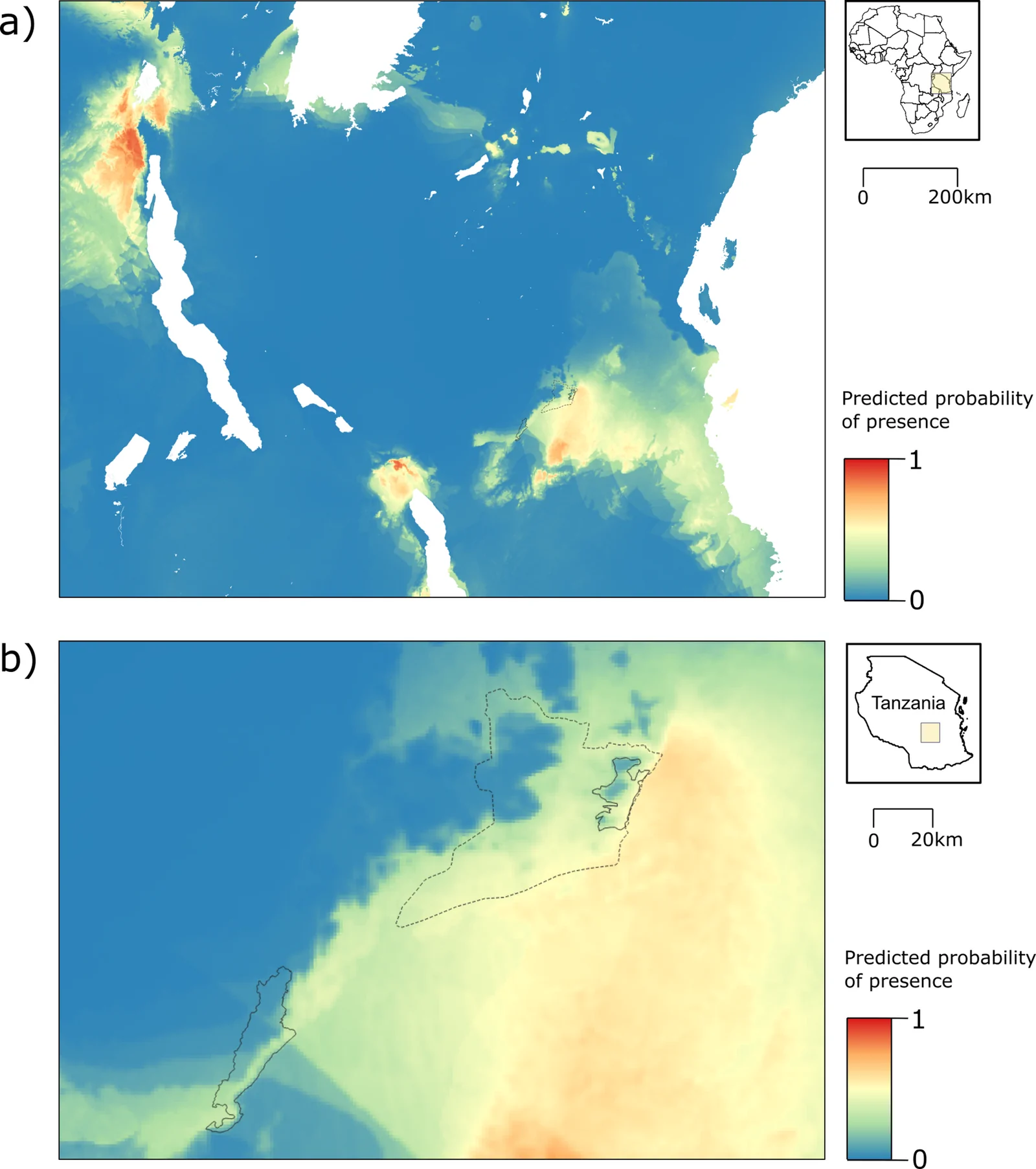
Estimated probability of habitat suitability for the Sanje mangabey (Cercocebus sanjei) within the Udzungwa Mountains during the Last Interglacial period (~120,000–140,000 years ago) across (**a**) Tanzania and (**b**) the Udzungwa Mountains

In the present-day scenario, the estimated suitable habitat and probability of presence is high (> 0.7) across southern Tanzania, extending from the Southern Highlands across to the southern region of the Udzungwa Mountains (Fig. 3). In the Mid-Holocene (~ 6,000 YA), the estimated suitable habitat is larger than the current scenario, extending over most of south Tanzania and covering the majority of the Udzungwa Mountain range (Fig. 4). During the Last Glacial Maximum (~ 22,000 YA), the estimated suitable habitat was smaller compared to that shown in the mid-Holocene, resembling more the current day habitat suitability however high probability regions extended further south from the Southern Highlands (Fig. 5). Finally, in the Last Interglacial period (~ 120,000–140,000 YA), the estimated suitable habitat was even smaller, with fragments of suitable habitat north-west of the Tanzanian border and Lake Tanganyika, in the Southern Highlands and south of the Udzungwa Mountains in the Kilombero Valley (Fig. 6).

## Discussion

The two Sanje mangabey populations were found to be highly significantly differentiated, with no shared haplotypes. The most closely related haplotypes from each population had 16 mutational differences. Our results support the hypothesis of significant genetic differentiation between populations of the Sanje mangabey, which may have occurred on a more ancient timescale. The ecological niche modelling over the past 140,000 years suggested suitable habitat expanded and contracted between the modelled time frames for the current, Mid-Holocene (~ 6,000 YA), Last Glacial Maximum (~ 22,000 YA) and Last Interglacial period (~ 120,000–140,000) scenarios but we did not find evidence of changes in female effective population size. Continuous suitable bioclimatic conditions appear to be present between the two forest fragments in which the extant Sanje mangabey populations are found. Our results suggest that factors other than bioclimatic may have limited dispersal, potentially leading to the observed genetic differentiation.

This was the first study to estimate the genetic diversity of the Sanje mangabey. The mitochondrial haplotype and nucleotide diversity were much lower in Mwanihana (*h* = 0.056 ± 0.052; π = 0.00013 ± 0.00012) than in Uzungwa Scarp (*h* = 0.664 ± 0.055; π = 0.00417 ± 0.00041). It is unknown if this genetic pattern is common since there are no comparable studies for other *Cercocebus* species for population diversity for the mitochondrial control region. Compared to population genetic studies of other Papionin species (Table 4), the Mwanihana Sanje mangabey population shows some of the lowest levels of nucleotide and haplotype diversity; however, the Uzungwa Scarp diversity was more reflective of the other studies (Table 4). Haplotype diversity in Mwanihana is likely representative of the diversity present in the population as the most frequent haplotype was sampled at all eleven independent sites, covering most of the distribution in that forest. In the Uzungwa Scarp, the number of haplotypes increased with increased sampling locations throughout the study. Further, the similar frequency of each of the four haplotypes present suggests that the diversity may increase with further sampling. For example, one haplotype was present in the far north and south of the Uzungwa Scarp and yet had 3 mutational differences to the next most closely related haplotype. This may be reflective of the diversity present, but more likely suggests that intermediates between these two haplotypes occur across the forest (Fig. 2). Although the diversity in the Uzungwa Scarp may increase with further sampling, the differentiation between populations is likely to reflect the result in this study.

**Table 4:** Comparison of nucleotide and haplotype diversity found in this study for the two populations of the Sanje mangabey (*Cercocebus sanjei*): Mwanihana (MW) and Uzungwa Scarp (US), with other Papionin species for fragments of the hypervariable (HV) regions of the mitochondrial control region

Species	Fragment	Nucleotide diversity	Haplotype diversity	Reference
*Cercocebus sanjei*				
MW	Partial HVI and HVII	0.00013	0.056	This study
US		0.00417	0.664	
*Theropithecus gelada*	1800 bp mtDNA incl. HVI (462 bp)	0.003298 (0.00062–0.00732)	0.7332 (0.462–0.962)	Zinner et al. (2018a)
*Papio ursinus griseipes*	HVI (352 bp)	0.047533 (0.036–0.086)		Burrell (2008)
*Papio ursinus griseipes*	HVI (490 bp)	0.0048 ± 0.00098	0.611 ± 0.0019	Ferreira da Silva et al. (2025)
*Macaca assamensis*	HVI (complete)	0.0118	0.962	Khanal et al. (2018)
*Macaca fascicularis*	HVI (complete)	0.042 (0.00025–0.084)	0.73 (0.12–0.99)	Badhan et al. (2015)

All species included show male-biased dispersal to be comparable with that of the Sanje mangabey

We did not expect to find a higher mitochondrial haplotype and nucleotide diversity in the Uzungwa Scarp than in Mwanihana, nor to find that the Uzungwa Scarp diversity was high and comparable to other species (Table 4). Historically, Mwanihana has had greater protection than the Uzungwa Scarp, having been protected by National Park regulations since 1992. It was therefore expected that the population in Mwanihana would have experienced a lower decrease in effective population size and genetic diversity due to anthropogenic activities. Estimates of demographic parameters in each forest varied between past studies which has been justified by differences in methodologies (Greco et al. 2023). Group densities and census size estimates ranged from being relatively similar between forests with an inferred population size decrease in both populations over the last 20 years (Mwanihana: *N* = 1,712 individuals and Uzungwa Scarp: *N* = 1,455 individuals; Paddock et al. 2020 using acoustic distance sampling data) to Mwanihana showing higher average group density than Uzungwa Scarp (0.26 vs. 0.24 groups/km^2^, respectively) which was justified by a stronger impact of anthropogenic pressures at Uzungwa Scarp for the last decades (Greco et al. 2023 integrating camera-trapping and acoustic data). When inferring the demographic history of the populations, this study found no evidence for changes in the effective population size, evidence for bottlenecks or loss of genetic diversity for each population, on a recent anthropogenic timescale. Our inferences of the demographic trajectory using Tajima’s D, Fu’s F_S_, and BSP, suggest a stable female effective size for both areas. The current levels of genetic diversity found in each population probably reflect a period of isolation on an ancient time scale rather than being the result of recent anthropogenic factors (e.g., deforestation, hunting, etc.).

The significantly low number and diversity of mitochondrial haplotypes found in Mwanihana could represent a founder effect of a single female haplotype with the low diversity being maintained by the philopatric behaviour of females (Fernández 2017; McCabe and Thompson 2013). The star shape phylogeny obtained, with the central haplotype likely ancestral to a descendant rare haplotype segregated by a single mutation, suggests the population may be expanding, with a mutation presenting new diversity to the population, or that an expansion occurred previously but other rare haplotypes were lost (Ferreri et al. 2011). Nuclear genome-wide evidence is required to corroborate this hypothesis. Low mitochondrial diversity combined with a low nuclear diversity could suggest a more historically lower effective population size. For example, in the white-headed langur (*Trachypithecus leucocephalus*) both mitochondrial and nuclear DNA diversity were low, suggesting a low effective size, likely due to a local founder event and a consequent ‘shallow’ evolutionary history, strongly influenced by recent declines due to anthropogenic activities (Wang et al. 2019). Given use of a small number of markers can produce a biased result (Rakotoarivelo et al. 2019), a population level study of nuclear DNA diversity will help to further assess the phylogeographic history of the Sanje mangabey populations (Rakotoarivelo et al. 2019).

The two Sanje mangabey populations, separated by approximately 100 km, were found to differ significantly, with 95.49% of mitochondrial haplotype variation partitioned by population. This difference resembles the pattern found in other mammals, including primates. For the sympatric Udzungwa red colobus (*P. gordonorum*), Ruiz-Lopez et al. (2016) study using microsatellite markers revealed the Udzungwa Scarp population as the most genetically differentiated, while also exhibiting the highest proportion of private alleles among the five forest populations analysed (Mwanihana, Ndundulu, Matundu, Magombera, and Udzungwa Scarp; Fig. 1). The Uzungwa Scarp is presently in closer proximity to other forest blocks in the Udzungwa Mountains (Fig. 1) and it is expected that primates inhabiting this forest patch would have had a greater connectivity and dispersal opportunities. However, in the study by Ruiz-Lopez et al. (2016), it was estimated that gene flow may have occurred through Matundu, Ndundulu and Mwanihana and in contrast, Uzungwa Scarp and Magombera were found to be individual distinct units with restricted gene flow to these forests. As such, the colobus populations inhabiting Uzungwa Scarp and Magombera forests were proposed to be representative of ancient lineages and that the current anthropogenic barriers to dispersal (e.g., towns/villages shown in Fig. 1) may be maintaining the pre-existing dispersal barriers and genetic distinctiveness (Ruiz-Lopez et al 2016). Additionally, Bowkett et al. (2015) found significant pairwise genetic differentiation in both microsatellite and mitochondrial DNA between populations of Harvey’s duiker (*Cephalophus harveyi*) in the Uzungwa Scarp and Mwanihana, despite the presence of other populations throughout the Udzungwa Mountains. The potential explanation for this pattern was that major rivers had acted as barriers to duiker dispersal. Those rivers identified in the duiker study as potential dispersal barriers (Bowkett et al. 2015), namely the Mngeta, Ruipa and Lumemo Rivers, are likely to have also impacted the Sanje mangabey as they are all found between Uzungwa Scarp and Mwanihana (Fig. 1).

In the modelling of the current bioclimatic conditions and presence of Sanje mangabey, the most important environmental variables were a low range of isothermality (low levels of temperature variability from day to night and between seasons), moderate precipitation during the driest month and high annual precipitation. This is consistent with habitat requirements of stable forests which reflects the current distribution of the Sanje mangabey and the estimated historic dispersal patterns in line with forest habitat expansion and retraction. As the Sanje mangabey is considered flexible in its use of primary and secondary forest, and elephant-disturbed shrubland (McCabe and Thompson 2013), the populations may have successfully persisted in changing habitats over the recent past.

Ecological niche models for the Sanje mangabey indicate that suitable habitat has been continuously available throughout the southern Udzungwa Mountains and across all time periods examined e.g., current (1960–1990), Mid-Holocene (MH; ~ 6,000 YA), Last Glacial Maximum (LGM; ~ 22,000 YA), and Last Interglacial (LIG; ~ 120,000–140,000 YA) (Figs. 3, 4, 5, 6). This area encompasses the Udzungwa Scarp and Mwanihana forests, where extant populations persist, as well as the Kilombero Valley and the intermediate Matundu forest (Fig. 1), suggesting that bioclimatic conditions suitable for dispersal were maintained over time. Despite this apparent climatic stability, we detected strong genetic differentiation between the two Sanje mangabey populations. These results therefore indicate that factors other than bioclimatic ones, such as habitat structure, landscape configuration, and physical barriers, likely constrained dispersal, leading to the observed genetic differentiation. Our results also suggest the former presence of intermediate populations in other forest blocks that have since gone extinct, consistent with the hypothesis of a historically broader distribution across the Kilombero Valley lowland forests proposed by Homewood and Rodgers (1981).

The model’s results highlight the ability of biodiversity within the Eastern Arc Mountain forests to withstand global climatic changes (Fjeldsaå and Lovett 1997; Marchant et al. 2007; Mumbi et al. 2008). This point, combined with evidence for the long-term stability in Sanje mangabey effective population size during this time, suggests the species’ resilience to significant climatic fluctuations. A general drying trend and aridification events in the region have been estimated since 1.86 MYA (Trauth et al. 2009). Studies of hominin evolution in East Africa showed the climate becoming dryer 1.6–1.8 MYA, coinciding with glacial cycles (with the last cycle 1 MYA) and with evidence of a shift from closed canopy forest to grassland (de Menocal 2004). The climate also fluctuated between relative extremes during glacial and interglacial cycles, ~ 41 KY cycles from 2.8 MYA to ~ 100 KY cycles after 1.0 MYA, with this overall aridification pattern broadly continuing to the present day (de Menocal 2004). The models here appear to reflect a level of fluctuation over the last 140,000 years, with estimated suitable conditions expanding and contracting between time points. Therefore, in the case of the Sanje mangabey, this may have resulted in a general trend of forest habitat contraction over, and prior to, the time period modelled in this study, resulting in fragmentation and isolation of populations. Although not significant, female effective population size in the Uzungwa Scarp increased slightly since the Last Glacial Maximum (22,000 YA) (Fig. 1S, supplementary material). This may be reflected in the niche models showing more suitable habitat since that time point, with a large increase during the Mid-Holocene, in the Scarp/south-west region allowing the population to expand in that time.

Upward shifts of montane forest to higher elevations may represent an additional mechanism contributing to the genetic differentiation between Sanje mangabey populations. Analyses of a sediment core collected from a swamp near Iringa, located on the north-west boundary of the Udzungwa Mountains, estimated a shift in elevation of montane forests from 1,700–1,800 m 38 KYA, from 1850–1950 m 29 − 10 KYA and then to 2000 m 3.5 KYA to the present day (Mumbi et al. 2008). Although mangabeys are currently commonly found below 1,700 m, such elevational shifts of montane forests may have reduced and fragmented suitable habitat, even under climatically favourable conditions, increasing extinction risk for small, isolated populations and creating effective barriers to dispersal. Consistent with this, extant Sanje mangabey groups show higher occupancy farther from forest edges (Rovero et al. 2017), suggesting sensitivity to habitat fragmentation and edge effect. Over the last 3 MY, sympatric members of *Cercopithecus* have also likely been subject to forest oscillations, having become restricted to small and isolated refugia after 0.8 MYA (Butynski and Jong 2020), a similar time frame to the TMRCA between Sanje mangabey populations (0.77 MYA, Paddock et al. 2026). The gradual upward displacement of montane forests (Mumbi et al. 2008) may therefore have fragmented once-continuous habitats into isolated “islands,” driving local extinctions in intermediate forest blocks. Subsequent phases of forest expansion would then have failed to restore connectivity, as intermediate populations were no longer present to facilitate dispersal.

In addition, the contraction of suitable habitat may have led to greater competition for resources with sympatric primates that may have been detrimental to Sanje mangabey, which may have contributed to a decrease in dispersal between forest habitats at a time the forests were becoming smaller and fewer, contributing to further isolating Sanje mangabey populations (Paddock et al. 2026). Sympatric primates competing with the Sanje mangabey may have included yellow baboons, Sykes’ monkey (*C. m. albogularis*) and colobines, that live in association with the mangabey presently, and may have previously overlapped and competed with the kipunji. For instance, Sanje mangabeys have been observed avoiding yellow baboons (D. Fernández, Pers. Obs.). Although yellow baboons are rare in forests regardless of their elevation, they are currently found in higher densities at low elevations, and this may have prevented mangabeys from dispersing through the low elevation corridors (Paddock et al. 2026). Other mangabeys, such as the Tana River mangabey (*Cercocebus galeritus)* endemic to southeastern Kenya, have also been observed avoiding yellow baboons in areas of sympatry, with reduced within-group dispersal under high habitat overlap and displacement from sleeping sites (Wahungu 1998). However, it remains unclear when this proposed competition may have begun in the Udzungwa Mountains, and whether presence of yellow baboons predates the estimated timing of divergence between the two mangabey subpopulations. Previous studies showed that yellow baboons from the southern Udzungwa Mountains carry mtDNA haplotypes closely related to the sympatric kipunji, with an estimated divergence of 3.03 MY (CI: 2.06–4.09) from the remaining *Papio* lineages (Supplementary Fig. 1 and Table 2 in Zinner et al. 2018b). These haplotypes likely reflect inverted intergeneric introgression (Zinner et al. 2018b). In contrast, yellow baboons from the northern Udzungwa Mountains cluster with conspecifics of the northern yellow baboon clade, with an estimated divergence of ~ 0.10 MY (Supplementary Fig. 1a in Zinner et al. 2018b), which postdates the inferred divergence between the two Sanje mangabey subpopulations. The hypothesis that competition with yellow baboons has limited dispersal in Sanje mangabey requires further investigation.

### Conservation implications

Our results suggest an ancient timescale of significant mitochondrial differentiation between populations and therefore that the two populations may represent evolutionary distinct lineages. Primate studies increasingly recommend that ESUs be managed independently in conservation planning (e.g., Chaves et al. 2019; Mekonnen et al. 2018). Protecting genetic diversity and evolutionary potential of a species with a complex distribution can be challenging, and in the case of the Sanje mangabey, this means protecting two locations harboring different levels of genetic diversity and with different protection needs. Our study implies that the formal protection of Uzungwa Scarp should be improved since most of the genetic diversity of the species is present in this forest.

As with the Udzungwa red colobus (Ruiz-Lopez et al. 2016), it is estimated that differentiation and isolation of the two sub-populations of Sanje mangabey has occurred on an ancient timescale; however, the more immediate threats to the species are the anthropogenic barriers to dispersal, including further degradation of suitable habitat. Work to establish corridors between forest fragments, such as the Mngeta corridor connecting the Uzungwa Scarp to Matundu and the Kilombero Nature Reserve (Rovero and Jones 2012), is ongoing; however, these forests are at lower elevations and may not be suitable for Sanje mangabey dispersal and/or occupation. In addition, negative interactions between mangabeys and other primates (possibly yellow baboons) may have prevented mangabeys from dispersing through the low elevation corridors. One solution is to promote regeneration and extension of both Uzungwa Scarp and Mwanihana forests at higher elevations, an undertaking that would require significant resources. In any case, immediate action is needed to reduce habitat loss, fragmentation and poaching in both populations, with standardized monitoring and effective area management, particularly in the Udzungwa Scarp Forest Reserve, required to reduce further loss of genetic diversity for the species. This work should be closely integrated with inclusive, community-led conservation initiatives that help reduce existing tensions between local communities and protected areas, while generating tangible benefits for those communities (Dempsey et al. 2024).

## Supplementary Information

Below is the link to the electronic supplementary material.

Supplementary Material 1

## Data Availability

Nucleotide sequence data reported are available in the GenBank databases under the accession numbers  – PV933218 to PV933281.

## References

[CR2] Bertola LD, Brüniche-Olsen A, Kershaw F, Russo I-RM, MacDonald AJ, Sunnucks P et al (2024) A pragmatic approach for integrating molecular tools into biodiversity conservation. Conserv Sci Pract 6(1):e13053. 10.1111/csp2.13053

[CR3] Bouckaert R, Vaughan TG, Barido-Sottani J, Duchêne S, Fourment M et al (2019) BEAST 2.5: an advanced software platform for Bayesian evolutionary analysis. PLoS Comput Biol 15:e1006650. 10.1371/journal.pcbi.1006650 30958812 PMC6472827

[CR4] Bowkett AE, Jones T, Rovero F, Nielsen MR, Plowman AB, Stevens JR (2015) Genetic patterns in forest antelope populations in the Udzungwa Mountains, Tanzania, as inferred from non-invasive sampling. J East Afr Nat Hist 104:91–125 (**35**)

[CR5] Butynski TM, Jong YAD (2020) Taxonomy and biogeography of the gentle monkey *Cercopithecus mitis* Wolf, 1822 (Primates: Cercopithecidae) in Kenya and Tanzania, and designation of a new subspecies endemic to Tanzania. Primate Conserv 34:71–127

[CR6] Chaves PB, Magnus T, Jerusalinsky L, Talebi M, Strier KB et al (2019) Phylogeographic evidence for two species of muriqui (genus *Brachyteles*). Am J Primatol 81:e23066. 10.1002/ajp.23066 31736121

[CR7] Darriba D, Taboada GL, Doallo R, Posada D (2012) jModelTest 2: more models, new heuristics and parallel computing. Nat Meth 9:772–772. 10.1038/nmeth.2109 PMC4594756 22847109

[CR8] de Menocal PB (2004) African climate change and faunal evolution during the Pliocene-Pleistocene. Earth Planet Sci Lett 220:3–24. 10.1016/S0012-821X(04)00003-2

[CR9] Dempsey A, Fernández DGM, Abernethy K, Abwe EE, et al. (2024) *Cercocebus* and *Mandrillus* conservation action plan 2024–2028. IUCN, Gland. Available via: https://iucn.org/resources/publication/cercocebus-and-mandrillus-conservation-action-plan-2024-2028

[CR10] Dinesen L, Lehmberg T, Rahner MC, Fjeldså J (2001) Conservation priorities for the forests of the Udzungwa Mountains, Tanzania, based on primates, duikers and birds. Biol Conserv 99:223–236. 10.1016/S0006-3207(00)00218-4

[CR12] Elith J, Phillips SJ, Hastie T, Dudík M, Chee YE, Yates CJ (2011) A statistical explanation of MaxEnt for ecologists. Divers Distrib 17:43–57. 10.1111/j.1472-4642.2010.00725.x

[CR13] Excoffier L, Lischer HE (2010) Arlequin suite ver. 3.5: a new series of programs to perform population genetics analyses under Linux and Windows. Mol Ecol Resour 10:564–567. 10.1111/j.1755-0998.2010.02847.x 21565059

[CR14] Fernández D (2017) Consequences of a male takeover on mating skew in wild Sanje mangabeys. Am J Primatol 79:e22532. 10.1002/ajp.22532 26822336

[CR15] Ferreira da Silva MJ, Bruford MW (2017) Genetics and primate conservation. In: Fuentes A, (ed) The international encyclopedia of primatology. Wiley

[CR16] Ferreira da Silva MJ, Tralma P, Colmonero-Costeira I, Cravo-Mota M, Farassi R et al (2025) Sex-mediated gene flow in grayfoot chacma baboons (*Papio ursinus griseipes*) in Gorongosa National Park, Mozambique. Int J Primatol 46:705–736. 10.1007/s10764-025-00494-2

[CR17] Ferreri M, Qu W, Han B (2011) Phylogenetic networks: a tool to display character conflict and demographic history. Acad J 10:12799–12803. 10.5897/AJB11.010

[CR18] Fitzgerald M, Coulson R, Lawing AM, Matsuzawa T, Koops K (2018) Modeling habitat suitability for chimpanzees (*Pan troglodytes verus*) in the Greater Nimba Landscape, Guinea, West Africa. Primates 59:361–375. 10.1007/s10329-018-0657-8 29524002

[CR19] Fjeldsaå J, Lovett JC (1997) Geographical patterns of old and young species in African forest biota: the significance of specific montane areas as evolutionary centres. Biodivers Conserv 6:325–346. 10.1023/A:1018356506390

[CR20] Frankham R (2010) Challenges and opportunities of genetic approaches to biological conservation. Biol Conserv 143:1919–1927. 10.1016/j.biocon.2010.05.011

[CR121] Greco I, Paddock CL, McCabe GM, Barelli C, Shinyambala S, Mtui AS, Rovero F (2023) Calibrating occupancy to density estimations to assess abundance and vulnerability of a threatened primate in Tanzania. Ecosphere 14(3):e4427. 10.1002/ecs2.4427

[CR21] Gregory SD, Brook BW, Goossens B, Ancrenaz M, Alfred R et al (2012) Long-term field data and climate-habitat models show that orangutan persistence depends on effective forest management and greenhouse gas mitigation. PLoS One 7:e43846. 10.1371/journal.pone.0043846 22970145 PMC3436794

[CR22] Heller R, Chikhi L, Siegismund HR (2013) The confounding effect of population structure on Bayesian skyline plot inferences of demographic history. PLoS One 8:e62992 23667558 10.1371/journal.pone.0062992 PMC3646956

[CR23] Homewood KM, Rodgers WA (1981) A previously undescribed mangabey from Southern Tanzania. Int J Primatol 2:47–55. 10.1007/BF02692299

[CR24] Jones T, Ehardt CL, Butynski TM, Davenport TRB, Mpunga NE et al (2005) The highland mangabey *Lophocebus kipunjii*: a new species of African monkey. Science 308:1161–1164. 10.1126/science.1109191 15905399

[CR25] Kass JM, Muscarella R, Galante PJ, Bohl CL, Pinilla-Buitrago GE et al (2021) ENMeval 2.0: redesigned for customizable and reproducible modeling of species’ niches and distributions. Methods Ecol Evol 12:1602–1608

[CR26] Khanal L, Chalise MK, He K, Acharya BK, Kawamoto Y, Jiang X (2018) Mitochondrial DNA analyses and ecological niche modeling reveal post-LGM expansion of the Assam macaque (*Macaca assamensis*) in the foothills of Nepal Himalaya. Am J Primatol 80:e22748 29536562 10.1002/ajp.22748

[CR27] Lawson LP, Vernesi C, Ricci S, Rovero F (2013) Evolutionary history of the grey-faced sengi, *Rhynchocyon udzungwensis*, from Tanzania: a molecular and species distribution modelling approach. PLoS One 8:e72506. 10.1371/journal.pone.0072506 24015252 PMC3754996

[CR28] Leigh JW, Bryant D (2015) Popart: full-feature software for haplotype network construction. Methods Ecol Evol 6:1110–1116. 10.1111/2041-210X.12410

[CR29] Liu C, Berry PM, Dawson TP, Pearson RG (2005) Selecting thresholds of occurrence in the prediction of species distributions. Ecography 28:385–393

[CR30] Liu J, Fitzgerald M, Liao H, Luo Y, Jin T et al (2020) Modeling habitat suitability for Yunnan snub-nosed monkeys in Laojun Mountain National Park. Primates 61:277–287. 10.1007/s10329-019-00767-4 31602589

[CR31] Lovett JC, Pocs T (1993) Assessment of the condition of the catchment forest reserves: a botanical appraisal. Dar es Salaam: Government Printers, pp. 300

[CR32] Marchant R, Mumbi C, Behera S, Yamagata T (2007) The Indian Ocean dipole – the unsung driver of climatic variability in East Africa. Afr J Ecol 45:4–16. 10.1111/j.1365-2028.2006.00707.x

[CR33] Marshall AR, Jørgensbye HI, Rovero F, Platts PJ, White PC, Lovett JC (2010) The species-area relationship and confounding variables in a threatened monkey community. Am J Primatol 72:325–336 20039329 10.1002/ajp.20787

[CR34] Marshall AR (2007) Disturbance in the Udzungwas: responses of monkeys and trees to forest degradation. University of York, York

[CR35] McCabe GM, Thompson ME (2013) Reproductive seasonality in wild Sanje mangabeys (*Cercocebus sanjei*), Tanzania: relationship between the capital breeding strategy and infant survival. Behaviour 150:1399–1429. 10.1163/1568539X-00003102

[CR36] McCabe GM, Fernández D, Ehardt CL (2013) Ecology of reproduction in Sanje mangabeys (*Cercocebus sanjei*): dietary strategies and energetic conditions during a high fruit period. Am J Primatol 75:1196–1208. 10.1002/ajp.22182 23897561

[CR37] McCabe GM, Rovero F, Fernández D, Butynski TM, Struhsaker TT (2019) *Cercocebus**sanjei*, IUCN. pp. e.T4203A17955753. Available: https://www.iucnredlist.org/es/species/4203/17955753

[CR38] Mekonnen A, Rueness EK, Stenseth NC, Fashing PJ, Bekele A et al (2018) Population genetic structure and evolutionary history of Bale monkeys (*Chlorocebus djamdjamensis*) in the southern Ethiopian Highlands. BMC Evol Biol 18:106. 10.1186/s12862-018-1217-y 29986642 PMC6038355

[CR39] Moritz C (1994) Defining ‘evolutionarily significant units’ for conservation. Trends Ecol Evol 9(10):373–375. 10.1016/0169-5347(94)90057-4 21236896

[CR40] Mumbi CT, Marchant R, Hooghiemstra H, Wooller MJ (2008) Late Quaternary vegetation reconstruction from the Eastern Arc Mountains, Tanzania. Quat Res 69:326–341. 10.1016/j.yqres.2007.10.012

[CR42] Otto-Bliesner BL, Marshall SJ, Overpeck JT, Miller GH, Hu A (2006) Simulating arctic climate warmth and icefield retreat in the last interglaciation. Science 311:1751–1753. 10.1126/science.1120808 16556838

[CR43] Paddock CL, Bruford MW, McCabe GM (2020) Estimating the population size of the Sanje mangabey (*Cercocebus sanjei*) using acoustic distance sampling. Am J Primatol 82:e23083. 10.1002/ajp.23083 31912545

[CR44] Paddock CL, Ferreira da Silva MJ, McCabe GM, Fernández D, McGraw WS, Bruford MW (2026) On the phylogenetic history of the Sanje mangabey (Cercocebus sanjei). Int J Primatol. 10.21203/rs.3.rs-5484999/v1 PMC13348350 42428162

[CR45] Phillips S. 2017. A Brief tutorial on maxent. Lessons Conserv 3: 108–135. Available via: https://www.amnh.org/content/download/141371/2285439/file/a-brief-tutorial-on-maxent.pdf

[CR47] Phillips SJ, Anderson RP, Schapire RE (2006) Maximum entropy modeling of species geographic distributions. Ecol Model 190:231–259. 10.1016/j.ecolmodel.2005.03.026

[CR48] R Core Team (2017) R: A language and environment for statistical computing. R Foundation for Statistical Computing (Vienna, Austria).

[CR49] Radespiel U, Bruford M (2014) Fragmentation genetics of rainforest animals: insights from recent studies. Conserv Genet 15:245–260. 10.1007/s10592-013-0550-3

[CR50] Rakotoarivelo AR, O’Donoghue P, Bruford MW, Moodley Y (2019) Rapid ecological specialization despite constant population sizes. PeerJ 7:e6476. 10.7717/peerj.6476 31041147 PMC6476403

[CR52] Rambaut A, Drummond AJ, Xie D, Baele G, Suchard MA (2018) Posterior summarization in Bayesian phylogenetics using Tracer 1.7. Syst Biol 67:901–904 29718447 10.1093/sysbio/syy032 PMC6101584

[CR53] Ramírez-Soriano A, Ramos-Onsins SE, Rozas J, Calafell F, Navarro A (2008) Statistical power analysis of neutrality tests under demographic expansions, contractions and bottlenecks with recombination. Genetics 179:555–567. 10.1534/genetics.107.083006 18493071 PMC2390632

[CR54] Roeder AD, Archer FI, Poinar HN, Morin PA (2004) A novel method for collection and preservation of faeces for genetic studies. Mol Ecol Notes 4:761–764

[CR55] Rovero F, Jones T (2012) Wildlife Corridors in the Udzungwa Mountains of Tanzania. Ecol Restor 30(4):282–285

[CR56] Rovero F, Luca DWD (2007) Checklist of mammals of the Udzungwa Mountains of Tanzania. Mammalia 71:47–55. 10.1515/MAMM.2007.015

[CR57] Rovero F, Marshall AR (2005) Diversity and abundance of diurnal primates and forest antelopes in relation to habitat quality: a case study from the Udzungwa mountains of Tanzania. Springer US, Boston

[CR58] Rovero F, Marshall AR, Jones T, Perkin A (2009) The primates of the Udzungwa Mountains: diversity, ecology and conservation. J Anthropol Sci 87:93–126 19663171

[CR60] Rovero F, Martin E, Rosa M, Ahumada JA, Spitale D (2014) Estimating species richness and modelling habitat preferences of tropical forest mammals from camera trap data. PLoS One 9:e103300 25054806 10.1371/journal.pone.0103300 PMC4108438

[CR62] Rovero F, Owen N, Jones T, Canteri E, Iemma A, Tattoni C (2017) Camera trapping surveys of forest mammal communities in the Eastern Arc Mountains reveal generalized habitat and human disturbance responses. Biodivers Conserv 26:1103–1119. 10.1007/s10531-016-1288-2

[CR63] Rozas J, Ferrer-Mata A, Sánchez-DelBarrio JC, Guirao-Rico S, Librado P et al (2017) DnaSP 6: DNA sequence polymorphism analysis of large data sets. Mol Biol Evol 34:3299–32302. 10.1093/molbev/msx248 29029172

[CR64] Ruiz-Lopez MJ, Barelli C, Rovero F, Hodges K, Roos C et al (2016) A novel landscape genetic approach demonstrates the effects of human disturbance on the Udzungwa red colobus monkey (*Procolobus gordonorum*). Heredity (Edinb) 116:167–176. 10.1038/hdy.2015.82 26374237 PMC4806883

[CR65] Struhsaker TT, Marshall AR, Detwiler K, Siex K, Ehardt C et al (2004) Demographic variation among Udzungwa red colobus in relation to gross ecological and sociological parameters. Int J Primatol 25:615–658. 10.1023/B:IJOP.0000023578.08343.4e

[CR66] Sunnucks P (2000) Efficient genetic markers for population biology. Trends Ecol Evol 15:199–203. 10.1016/s0169-5347(00)01825-5 10782134

[CR67] Thuiller W (2024) Ecological niche modelling. Curr Biol 34:R225–R229. 10.1016/j.cub.2024.02.018 38531309

[CR68] Townsend JP (2007) Profiling phylogenetic informativeness. Syst Biol 56:222–231. 10.1080/10635150701311362 17464879

[CR69] Trauth MH, Larrasoaña JC, Mudelsee M (2009) Trends, rhythms and events in Plio-Pleistocene African climate. Quat Sci Rev 28:399–411

[CR70] Wahungu G (1998) Diet and habitat overlap in two sympatric primate species, the Tana crested mangabey *Cercocebus galeritus* and yellow baboon *Papio cynocephalus*. Afr J Ecol 36:159–173

[CR71] Wang W, Zheng Y, Zhao J, Yao M (2019) Low genetic diversity in a critically endangered primate: shallow evolutionary history or recent population bottleneck? BMC Evol Biol 19:134. 10.1186/s12862-019-1451-y 31242851 PMC6595580

[CR73] WWF Tanzania Programme Office (2007) Workshop proceedings: conservation and management of the southern Udzungwa Mountains: the way forward (Dar Es Salaam: WWF-Tanzania Programme Office), pp. 29. Available via: http://www.udzungwacentre.org/documents/reports/cepf_march-07_workshop_proceedings-morogoro.pdf

[CR74] Zhang M-G, Zhou Z-K, Chen W-Y, Slik JWF, Cannon CH, Raes N (2012) Using species distribution modeling to improve conservation and land use planning of Yunnan, China. Biol Conserv 153:257–264. 10.1016/j.biocon.2012.04.023

[CR75] Zinner D, Atickem A, Beehner JC, Bekele A, Bergman TJ et al (2018a) Phylogeography, mitochondrial DNA diversity, and demographic history of geladas (*Theropithecus gelada*). PLoS One 13:e0202303 30138418 10.1371/journal.pone.0202303 PMC6107150

[CR76] Zinner D, Chuma IS, Knauf S, Roos C (2018b) Inverted intergeneric introgression between critically endangered kipunjis and yellow baboons in two disjunct populations. Biol Lett 14:20170729. 10.1098/rsbl.2017.0729 29343565 PMC5803601

