# Supplementary material for: Evolutionary history of the endangered Sanje mangabey (Cercocebus sanjei) in the Udzungwa Mountains, Tanzania: inferences from phylogeography and historical niche modelling
Source: Primates. 2026 Apr 10;67(5):483–99. doi: 10.1007/s10329-026-01255-2 (PMC13541865; doi:10.1007/s10329-026-01255-2)
Supplement: Supplementary file 1 [file 10329_2026_1255_MOESM1_ESM.docx]

Online material:

Evolutionary History of the Endangered Sanje Mangabey (Cercocebus sanjei) in the Udzungwa Mountains, Tanzania: Inferences from Phylogeography and Historical Niche Modelling

Paddock, et al.

**Section 1 – Primers, final concentration of reagents in PCR and PCR cycling conditions**

We amplified the fragment using sequences from two overlapping primers and the PCR cycling conditions indicated in Table 1. The amplified fragment is between 15,637 and 16,214 bp of voucher *Cercocebus atys* accession number KP090062.1).

| **Table 1.** Primer sequences to amplify two overlapping fragments of the mitochondrial control region for the Sanje mangabey (*Cercocebus sanjei*) from (Paddock, Ferreira da Silva et al. 2024) | | | |
| --- | --- | --- | --- |
| **Primers** | | **Fragment length (bp)** | **PCR Cycling Conditions** |
| CRF-1 | GCTCCGGGCCCATAACTC | 257 | 95°C for 2 mins, [95°C for 30s, 61.8°C for 60s, 72°C for 2 mins] x 35 cycles, 72°C for 10 mins |
| CRR-1 | CAAAGACAGGCGCATTCAGG |  |  |
| CRF-2 | CCRAAACATGCTTACAAGC | 343 | 95°C for 2 mins, [95°C for 30s, 50°C for 60s, 72°C for 2 mins] x 35 cycles, 72°C for 10 mins |
| CRR-2 | GTTATGGCCCTGAGGTAAG |  |  |

We amplified each fragment by PCR in a total volume of 10μl, consisting of 5μl Bioline MyTaq™, 1μl template DNA and forward and reverse primers at a final concentration of 2μM.

All PCR cycling programmes were run in an Applied Biosystems GeneAmp PCR System 9700.

The success of each PCR was tested by agarose gel electrophoresis and visualised in a UV transilluminator.

Several precautions were taken to avoid and verify the co-amplification of nuclear mitochondrial inserts (NUMTS), following recommendations of (Bensasson, Zhang et al. 2001):

i) sequence chromatograms were inspected for double peaks that would indicate the presence of sequences co-amplified by PCR and which could represent contaminations or NUMTS, ii) only a single PCR amplicon of the correct length was present, iii) any single base extreme variants found during the alignment of sequences were verified, and iv) four independent primers amplifying overlapping fragments were used, with the overlapping sequences matching from separate PCRs (Table 1).

**Table 2S.** Current bioclimatic variables (1960-1990) from WorldClim Version 1.4 (http://worldclim.org) analysed during ecological niche modelling for the Sanje mangabey in the Udzungwa Mountains, Tanzania. Variables used in the model after testing for autocorrelation are indicated in the final column.

| **Variable** | **Description** |  |  |
| --- | --- | --- | --- |
| Bio1 | Annual mean temperature |  | ✓ |
| Bio2 | Mean diurnal range: (Mean max. temp – min. temp) |  | ✓ |
| Bio3 | Isothermality: temperature variability from day to night and between seasons - (Bio2/Bio7) *100 |  | ✓ |
| Bio4 | Temperature seasonality: (Standard Deviation * 100) |  |  |
| Bio5 | Max. temperature warmest month |  |  |
| Bio6 | Min. temperature coldest month |  |  |
| Bio7 | Temperature annual range: (Bio5 – Bio6) |  |  |
| Bio8 | Mean temperature of wettest quarter |  |  |
| Bio9 | Mean temperature of driest quarter |  |  |
| Bio10 | Mean temperature of warmest quarter |  |  |
| Bio11 | Mean temperature of coldest quarter |  |  |
| Bio12 | Annual precipitation |  | ✓ |
| Bio13 | Precipitation of wettest month |  |  |
| Bio14 | Precipitation of driest month |  | ✓ |
| Bio15 | Precipitation seasonality |  |  |
| Bio16 | Precipitation of wettest quarter |  |  |
| Bio17 | Precipitation of driest quarter |  |  |
| Bio18 | Precipitation of warmest quarter |  | ✓ |
| Bio19 | Precipitation of coldest quarter |  |  |

**Figure. 1S** Bayesian Skyline Plots for a 423bp fragment of the mtDNA control region and the mean female effective population size for two populations of Sanje mangabey (*Cercocebus sanjei*) found in: a) Mwanihana, and b) Uzungwa Scarp forest. No change was detected in the Mwanihana population (Figure 2a) however the Uzungwa Scarp (Figure 2b) showed a slight increase in female effective population size from ~6,000 YA. The plot for Uzungwa Scarp (Figure 2b) shows two relevant time points from the historic ecological niche models in this study for reference, the Mid-Holocene (~6,000 YA) and the Last Glacial Maximum (~22,000 YA).

| **Table 3S.** The estimates of contributions to the Maxent model by each variable. | | | |
| --- | --- | --- | --- |
| **Variable** | | **Contribution (%)** | **Permutation importance** |
| bio3 | Isothermality | 38.8 | 36.4 |
| bio14 | Precipitation during the driest month | 38.5 | 52.4 |
| bio12 | Annual precipitation | 19.3 | 5.5 |
| bio1 | Annual mean temperature | 2.1 | 4.5 |
| bio2 | Mean diurnal range | 1.2 | 1.2 |
| bio18 | Precipitation of warmest quarter | 0 | 0 |

**Figure 2S.** Jackknife test of importance for each variable by testing the training gain of the model. The graph shows for each variable, the difference in average gain built without a given variable (dark pink) and models built with only that variable (green) using training and test data. The variables with smaller differences are ones most related to species distribution.

| a) |  |
| --- | --- |
| b) |  |
| c) |  |
| **Figure 3S.** Response curves, by logistic output, in the ecological niche model for the top three contributing environmental variables: a) isothermality, b) precipitation during the driest month, and c) annual precipitation. The dark grey line shows the mean logistic output of the probability of presence for each environmental variable and the shaded grey area ± one standard deviation. | |

**References**

Bensasson, D., D.-X. Zhang, D. Hartl and G. Hewitt (2001). "Mitochondrial pseudogenes: Evolution’s misplaced witnesses." Trends Ecol Evol **16**: 314–321.

Paddock, C. L., M. Ferreira da Silva, G. M. McCabe, D. Fernández, W. S. McGraw and M. W. Bruford (2024). "On the phylogenetic history of the Sanje mangabey (Cercocebus sanjei)." PREPRINT (Version 1) available at Research Square
